# Physiological effects of an oil rich in γ-linolenic acid on hepatic fatty acid oxidation and serum lipid levels in genetically hyperlipidemic mice

**DOI:** 10.3164/jcbn.18-64

**Published:** 2018-11-15

**Authors:** Takashi Ide, Izumi Origuchi

**Affiliations:** 1 Department of Food and Nutrition, Faculty of Human Life, Jumonji University, 2-1-28 Sugasawa, Niiza, Saitama 352-8510, Japan

**Keywords:** apolipoprotein E-deficient mice, γ-linolenic acid, hepatic fatty acid oxidation, hepatic lipogenesis, serum lipids

## Abstract

We investigated the physiological activity of an oil rich in γ-linolenic acid of evening primrose origin (containing 42.6% γ-linolenic acid) affecting hepatic fatty acid metabolism, and serum lipid levels in genetically hyperlipidemic mice deficient in apolipoprotein E expression. Male apolipoprotein E-deficient mice (BALB/c.KOR/StmSlc-*Apoe*^*shl*^) were fed experimental diets containing 100 g/kg of palm oil (saturated fat), safflower oil (rich in linoleic acid), γ-linolenic acid oil (rich in γ-linolenic acid), or fat mixtures composed of safflower and γ-linolenic acid oils (65:35 and 30:70, w/w) for 20 days. γ-Linolenic acid oil, compared with palm and safflower oils, strongly and dose-dependently increased the activity and mRNA levels of hepatic fatty acid oxidation enzymes. In general, safflower and γ-linolenic acid oils, compared with palm oil, reduced the activity and mRNA levels of lipogenic enzymes. However, these oils were equivalent in reducing the parameters of lipogenesis, excluding malic enzyme and pyruvate kinase. The diets containing safflower and γ-linolenic acid oils, compared with the palm oil diet, significantly decreased serum triacylglycerol and cholesterol levels. The decreases were greater with γ-linolenic acid oil than with safflower oil. γ-Linolenic acid oil exerted strong serum lipid-lowering effects in apolipoprotein E-deficient mice apparently through the changes in hepatic fatty acid metabolism.

## Introduction

Many studies have found that various types of polyunsaturated fatty acids (PUFAs) influence hepatic fatty acid oxidation and synthesis. Physiological activities of respective PUFA affecting hepatic fatty acid synthesis and oxidation differ considerably from each other. Regarding the physiological activities of γ-linolenic acid (GLA), a *n*-6 trienoic acid, one report stated that a fungal oil rich in GLA, compared with soybean oil, increased carnitine palmitoyltransferase activity and the peroxisomal β-oxidation rate in the rat liver.^(1)^ Furthermore, it has been demonstrated that borage oil rich in GLA, compared with a saturated fat (palm oil) and fats rich in linoleic acid but devoid of GLA, increases hepatic peroxisomal fatty acid oxidation,^(2,3)^ and the activities of carnitine palmitoyltransferase^(2,3)^ and acyl-CoA oxidase^(2)^ in rats. In addition, borage oil increased mRNA levels of some peroxisomal enzymes involved in hepatic fatty acid oxidation but was ineffective in increasing mRNA levels of mitochondrial fatty acid oxidation enzymes.^(3)^ Regarding the physiological activity of GLA affecting hepatic lipogenesis, dietary oil rich in GLA compared with palm oil reduced the activities of enzymes involved in lipogenesis in the rat liver. However, the reducing effects were comparable with those observed with an oil rich in linoleic acid but devoid of GLA.^(2)^ Studies have indicated that oils rich in GLA lower serum cholesterol^(1,4–6)^ and triacylglycerol levels in rats.^(2,7,8)^ It is probable that up-regulation of hepatic fatty acid oxidation is the mechanism underlying the hypolipidemic effects of oils rich in GLA. However, a considerable number of studies^(3,9,10)^ have failed to observe serum lipid-lowering effects of oils rich in GLA. Therefore, careful evaluation of lipid-lowering propensity of oils rich in GLA is still required. Up-regulation of uncoupling protein 1 in brown adipose tissue rather than of hepatic fatty acid oxidation may be the mechanism underlying the attenuation of body fat accumulation in rats fed oils rich in GLA.^(1,3)^

Previous studies using rats as experimental animals found that dietary GLA enhanced hepatic fatty acid oxidation, but its serum lowering propensity was controversial. Animal models of hyperlipidemia are useful for clarifying the physiological activity of dietary factors affecting serum lipid levels. In relation to this, Fan *et al.*^(11)^ evaluated the anti-atherogenic effects of evening primrose oil rich in GLA in apolipoprotein E (apoE) knock-out mice with C57BL/6 genetic background. They demonstrated that dietary GLA was effective in attenuating progression of atherosclerosis, but it did not lower serum lipid levels in apoE knock-out mice. There is the possibility that apoE knock-out mice with C57BL/6 genetic background are not suitable for evaluating the physiological activities of dietary PUFAs affecting serum lipid levels. Indeed, previous studies^(12,13)^ reported that fish oil abundant in eicosapentaenoic and docosahexaenoic acids (EPA and DHA) failed to affect serum lipid levels in apoE knock-out mice even though many animal experiments using rats^(14,15)^ and mice^(16–18)^ resulted in potent serum lipid lowering effects of fish oil or EPA and DHA. Given the above, we herein investigated the physiological activity of an oil rich in GLA of evening primrose origin that affects hepatic fatty acid metabolism and serum and liver lipid profiles using hyperlipidemic BALB/c.KOR/StmSlc-*Apoe*^*shl*^ mice deficient in the expression of apoE. The congenic apoE-deficient mice with the genetic background of BALB/c were established by transferring the apoE gene mutation found in Japanese wild mice of the KOR genetic background through repeated backcrossing. We demonstrated that *Apoe*^*shl*^ mice with the genetic background of BALB/c in contrast to apoE knock-out mice with C57BL/6 genetic background was useful for evaluating different effects of fish oil and EPA/DHA ethylesters affecting hepatic fatty acid metabolism and serum lipid profiles.^(19)^ We herein clarified that the oil rich in GLA of evening primrose origin is a potent inducer of hepatic fatty acid oxidation and that it markedly affects serum lipid levels in *Apoe*^*shl*^ mice.

## Materials and Methods

### Animals and diets

Male BALB/c.KOR/StmSlc-*Apoe*^*shl*^ mice were obtained from Japan SLC, Inc., Hamamatsu, Japan at 4–5 weeks of age. Mice were housed individually in suspended wire-bottomed stainless-steel cages in a room with controlled temperature (21–23°C), humidity (55–65%), and lighting (lights on from 07:00 to 19:00), and fed commercial chow. After 12 days of acclimatization, mice were randomly divided into five groups with equal mean body weight consisting of 7 animals each and fed purified experimental diets for 20 days. Experimental diets contained 100 g/kg of either palm oil, safflower oil, an oil rich in γ-linolenic acid (GLA oil), or fat mixtures composed of safflower and GLA oils (65:35 and 30:70, w/w). GLA oil containing 42.6% GLA prepared by the selective hydrolysis of evening primrose oil containing 10.5% GLA using lipase of *Candida cylindracea* origin was donated by Tama Biochemical Co., Ltd., Tokyo, Japan. The fatty acid compositions of the dietary fats are shown in Table 1. The purified experimental diets contained (g/kg): casein, 200; dietary fat, 100; corn starch, 150; cellulose, 20; mineral mixture,^(20)^ 35; vitamin mixture,^(20)^ 10; l-cystine, 3; choline bitartrate, 2.5 and sucrose, 479.5. Animals had free access to the diets and water during the experimental period until they were euthanized. This animal experiment was approved by the review board of animal ethics of Jumonji University (approval number 1510; issued on January 5, 2016), and we followed the University’s guidelines for the care and use of experimental animals.

### Enzyme assays

At the end of the experiment, mice were anesthetized by isoflurane and euthanized by bleeding from the inferior vena cava from 09:00 to 11:00, after which the livers were quickly excised. Approximately 0.5 g of each liver was homogenized in 5 ml of 0.25 M sucrose containing 1 mM EDTA and 3 mM Tris-HCl (pH 7.2), and 3 ml of the homogenates were centrifuged at 200,000 *g* for 30 min. The activities of enzymes involved in fatty acid oxidation and synthesis were measured using total homogenates and the 200,000 *g* supernatant of the liver homogenate, respectively.^(19)^

### RNA analyses

Liver RNA was extracted by the method of Chomczynski and Sacchi.^(21)^ The quantity and quality of the RNA were assessed by measuring absorbance at 260, 280, and 320 nm, and by electrophoresis on 1.0% agarose gels. mRNA abundance was measured using the SYBR Green real-time PCR method as described previously.^(22)^ The reaction specificity for this method was verified by a melting curve analysis. mRNA abundance was calculated as a ratio to the mRNA abundance of β-actin in each cDNA sample and expressed as a fold change, assigning a value of 1 for mice fed a diet containing 100 g/kg of palm oil.

### Analyses of lipids and carnitine

Liver lipids were extracted and purified, and triacylglycerol, phospholipid, and cholesterol concentrations in the lipid extract were determined as described previously.^(23)^ The serum high-density lipoprotein (HDL) fraction was prepared by precipitating apolipoprotein B-containing lipoproteins using polyethylene glycol (average molecular weight of 6,000, Sigma-Aldrich Japan Co., Tokyo, Japan).^(24)^ Serum triacylglycerol, cholesterol, phospholipid, and free fatty acid concentration, as well as cholesterol and phospholipid level in the serum HDL fraction were analyzed using commercial enzyme kits (Wako Pure Chemical, Osaka, Japan). Cholesterol and phospholipid concentrations in the very low- and low-density lipoprotein (VLDL + LDL) fractions were calculated by subtracting the values in the HDL fraction from those in unfractionated serum samples. The hepatic concentration of carnitine was analyzed by the method of Pearson *et al.*^(25)^

### Statistical analysis

Microsoft Excel add-in software (Excel Statistics 2015, Social Survey Research Information Co., Tokyo, Japan) was used for statistical analyses. Data were expressed as means and their standard errors. The constancy of the variance and normality of the distribution of the observations were evaluated using Levene’s test and the Kolmogorov–Smirnov test, respectively. If variances were heterogeneous and/or the distributions were not normal, they were transformed logarithmically. These transformations were successful in rendering the variance of the observations constant and the distribution of data normal, and hence the transformed values were used for subsequent statistical evaluations. Data were analyzed using one-way ANOVA and Tukey’s post-hoc test to evaluate significant differences of all means. Differences were considered significant when *p*<0.05.

## Results

### Growth parameters and liver weights

Fat types did not significantly affect food intake or growth among the groups (Table 2). Liver weights were the same between animals fed palm oil or safflower oil as the sole dietary fat source. GLA oil dose-dependently increased this value. All values in mice fed diets containing different levels of GLA oil were significantly higher than those in the animals fed diets containing palm or safflower oil as the sole dietary fat source.

### Activity and mRNA levels of enzymes involved in hepatic fatty acid oxidation

**Fig. 1:**
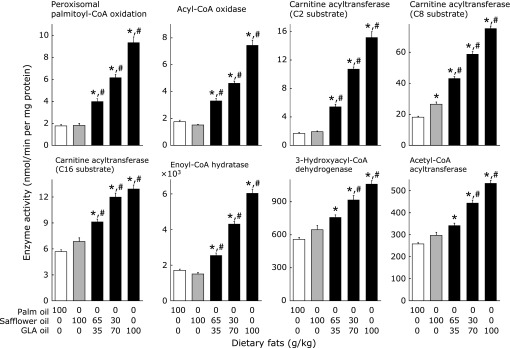
Effects of dietary fat on the activity of enzymes involved in fatty acid oxidation in the mouse liver. Carnitine acyltransferase activities were measured using three types of acyl-CoA substrates differing in carbon chain lengths (acetyl- (C2), octanoyl- (C8), and palmitoyl-CoAs (C16)). Values are mean ± SEM, *n* = 7. *p<0.05 vs mice fed 100 g/kg palm oil diet. ^#^*p*<0.05 vs mice fed 100 g/kg safflower oil diet. GLA oil, an oil rich in γ-linolenic acid.

A diet containing safflower oil as the sole dietary fat source, compared with palm oil diet, slightly but significantly increased the activity of carnitine acyltransferase measured using octanoyl-CoA as a substrate (Fig. 1). However, this diet, compared with palm oil diet, was ineffective in modifying the activities of other enzymes involved in fatty acid oxidation. GLA oil dose-dependently increased the activities of various enzymes involved in fatty acid oxidation. GLA oil, even at 35 g/kg, compared with the diet containing either palm or safflower oil as the sole dietary fat source, significantly increased the values, except for in two instances. The activities of 3-hydroxyacyl-CoA dehydrogenase and acetyl-CoA acyltransferase in mice fed the 35 g/kg GLA oil diet were not significantly different from those in animals fed a diet containing safflower oil as the sole dietary fat source.

**Fig. 2:**
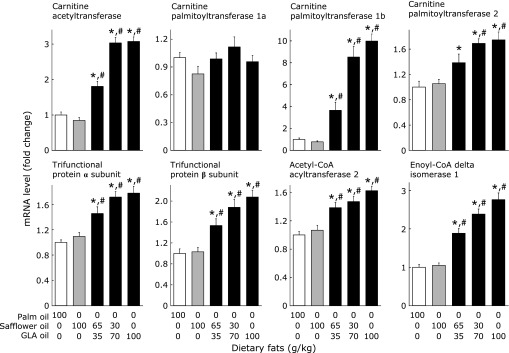
Effects of dietary fat on the mRNA levels of mitochondrial enzymes involved in fatty acid oxidation in the mouse liver. Values are mean ± SEM, *n* = 7. *p<0.05 vs mice fed 100 g/kg palm oil diet. ^#^*p*<0.05 vs mice fed 100 g/kg safflower oil diet. GLA oil, an oil rich in γ-linolenic acid.

Figure 2 shows the mRNA levels of mitochondrial fatty acid oxidation enzymes. Dietary GLA oil, even at 35 g/kg, compared with palm and safflower oils, significantly increased the mRNA levels of various mitochondrial fatty acid oxidation enzymes, except for carnitine palmitoyltransferase 1a. These values further increased as dietary levels of GLA oil increased. However, the response of the mRNA level of carnitine palmitoyltransferase 1a to dietary fats was considerably different from that of the other mitochondrial fatty acid oxidation enzymes. The type of dietary fat did not influence the mRNA levels of this enzyme.

**Fig. 3:**
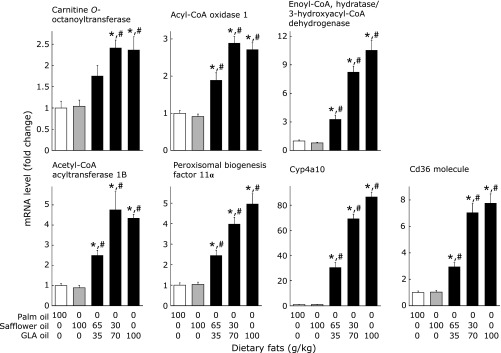
Effects of dietary fat on the mRNA levels of peroxisomal enzymes involved in fatty acid oxidation, peroxisomal membrane protein peroxin 11α, microsomal cyp4a10, and plasma membrane cd36 in the mouse liver. Values are mean ± SEM, *n* = 7. *p<0.05 vs mice fed 100 g/kg palm oil diet. ^#^*p*<0.05 vs mice fed 100 g/kg safflower oil diet. GLA oil, an oil rich in γ-linolenic acid.

Figure 3 shows mRNA levels of peroxisomal enzymes involved in hepatic fatty acid oxidation. The mRNA levels of the peroxisomal membrane protein, peroxisomal biogenesis factor 11α, as well as those of microsomal cyp4a10 involved in ω-oxidation of fatty acids and CD36 molecule, a plasma membrane protein that plays a role in the cellular uptake of fatty acids from the blood stream, are also shown in this figure. Again, GLA oil, compared with palm and safflower oils, caused large and dose-dependent increases in the mRNA levels of these proteins.

We also measured mRNA levels of peroxisome proliferator-activated receptor α (PPARα), but dietary fat-dependent changes were not observed. The values were 1.00 ± 0.07 and 0.956 ± 0.097 for mice fed diets containing palm or safflower oil as a sole fat source, respectively. The levels in mice fed diets containing varying concentrations of GLA oil were 1.11 ± 0.026, 1.21 ± 0.08, and 1.04 ± 0.06 for the animals fed diets containing 35, 70, and 100 g/kg GLA oil, respectively.

### Activity and mRNA levels of enzymes involved in hepatic lipogenesis

The activity levels of fatty acid synthase, ATP citrate lyase, glucose 6-phosphate dehydrogenase, and pyruvate kinase were lower in mice fed polyunsaturated fat diets than in those fed a palm oil diet (Table 3). GLA oil, compared with safflower oil, did not affect the activities of fatty acid synthase, ATP citrate lyase, or glucose 6-phosphate dehydrogenase. In contrast, GLA oil markedly decreased the activities of pyruvate kinase; these values in animals fed the diets containing 70 and 100 g/kg of GLA oil were less than one-half of those in animals fed a diet containing safflower oil as the sole fat source. The situation was considerably different for malic enzyme. A diet containing safflower oil as the sole fat source, compared with palm oil diet, significantly decreased the activity of malic enzyme. However, the values progressively increased as the dietary level of GLA oil increased. As a result, the activity level of this enzyme in mice fed a diet containing GLA oil as the sole fat source became comparable to that observed with a palm oil diet.

Table 4 shows the mRNA levels of proteins related to hepatic lipogenesis. There are two types of acetyl-CoA carboxylase, α and β. The α, but not β form, functions in long-chain fatty acid synthesis in the cytosol. Several isoforms of pyruvate kinase and malic enzyme have been identified in mammals. Pyruvate kinase liver and red blood cell (Pklr) and malic enzyme 1 encode the enzymes involved in hepatic lipogenesis. Glucose-6-phosphate dehydrogenase X-linked (G6pdx) encodes glucose 6-phosphate dehydrogenase. Experimental diets containing safflower and/or GLA oils, compared with a palm oil diet, decreased the mRNA levels of acetyl-CoA carboxylase α, fatty acid synthase, G6pdx, and Pklr. However, GLA oil and safflower oils reduced these values to a similar extent, except for Pklr mRNA levels. A diet containing safflower oil as the sole dietary fat source caused a 30% decrease in mRNA levels of Pklr compared with a palm oil diet. Replacement of safflower oil by GLA oil caused progressive additional decreases in the value. Responses of malic enzyme 1 mRNA levels to dietary fats were considerably different from those of other lipogenic enzymes. A diet containing safflower oil as the sole fat source, compared with a palm oil diet, significantly decreased this value. However, replacement of safflower oil by GLA oil dose-dependently increased the value. Consequently, the values observed with the diets containing 70 and 100 g/kg of GLA oil became comparable to those in the animals fed a palm oil diet. We also measured the mRNA levels of enzymes involved in the desaturation of fatty acids (stearoyl-CoA desaturase 1, and fatty acid desaturase 1 and 2). The responses of these mRNA levels to dietary fats resembled those observed for the mRNA levels of malic enzyme 1. A safflower oil diet, compared with a palm oil diet, significantly decreased these values. However, the replacement of safflower oil by GLA oil dose-dependently increased the values. As a result, the values in mice fed diets containing 100 g/kg of GLA oil were similar to or even significantly higher than those in mice fed a palm oil diet. We also measured mRNA levels of transcription factors involved in the regulation of lipogenesis. Although the type of dietary fat did not affect the mRNA levels of sterol regulatory element binding transcription factor 1 variant 2, alternatively known as sterol regulatory element binding protein-1c (SREBP-1c) or carbohydrate response element binding protein α (ChREBPα), polyunsaturated fats compared with palm oil significantly reduced the mRNA levels of ChREBPβ. However, the values were the same among mice fed diets containing safflower oil alone or containing varying amounts of GLA oil.

### Hepatic carnitine concentrations, and mRNA levels of proteins involved in carnitine transport and synthesis

**Fig. 4:**
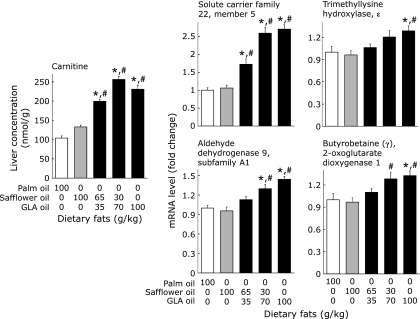
Effects of dietary fat on carnitine concentrations, and the mRNA levels of carnitine transporter and enzymes involved in carnitine biosynthesis in the mouse liver. Values are mean ± SEM, *n* = 7. *p<0.05 vs mice fed 100 g/kg palm oil diet. ^#^*p*<0.05 vs mice fed 100 g/kg safflower oil diet. GLA oil, an oil rich in γ-linolenic acid.

Diets containing GLA oil compared with diets containing palm or safflower oil as the sole fat source significantly increased the hepatic carnitine concentration (Fig. 4). Changes in hepatic carnitine concentrations by dietary fats paralleled those in the mRNA levels of carnitine transporter (solute carrier family 22, member 5), which mediates carnitine transport across the plasma membrane. Although the changes were attenuated, diets containing GLA oil, compared with diets containing palm or safflower oil as the sole fat source, increased the mRNA levels of genes encoding enzymes involved in carnitine biosynthesis (trimethyllysine hydroxylase, ε, aldehyde dehydrogenase 9, subfamily A1, and butyrobetaine (γ), 2-oxoglutarate dioxygenase 1, and the values for all three enzymes were significantly higher in mice fed the 100 g/kg GLA oil diet than in those fed the diet containing 100 g/kg of palm or safflower oil as the sole fat source.

### Serum and liver concentrations of lipids

The diets containing polyunsaturated fats (safflower and GLA oils), compared with a palm oil diet, significantly decreased serum triacylglycerol levels (Table 5). Decreases were greater with GLA oil than with safflower oil. Indeed, the levels were significantly lower in animals fed diets containing 70 or 100 g/kg of GLA oil than in those fed a diet containing 100 g/kg of safflower oil as a sole fat source. All polyunsaturated fat diets, compared with a palm oil diet, also significantly decreased serum cholesterol levels. In addition, the levels were lower in animals fed diets containing 35–100 g/kg of GLA oil than in those fed a diet containing 100 g/kg of safflower oil as the sole fat source. Cholesterol levels in the HDL fraction were the same between mice fed the diets containing palm or safflower oil as the sole fat source. However, diets containing varying amounts of GLA oil, compared with the diets containing palm or safflower oil as the sole fat source, significantly increased the HDL-cholesterol level. Conversely, the diets containing GLA oil, compared with palm or safflower oil diets, markedly reduced cholesterol concentrations in the fraction containing both VLDL and LDL. Again, polyunsaturated fat diets, compared with a palm oil diet, significantly decreased the serum phospholipid concentration. However, all the diets containing GLA oil, compared with a diet containing 100 g/kg of safflower oil as the sole fat source, failed to affect this value. HDL-phospholipid levels were similar between animals fed diets containing palm oil or safflower oil as the sole fat source. All diets containing GLA oil, compared with diets containing palm or safflower oil as the sole fat source, significantly increased the values. The diets containing safflower and GLA oils, compared with a palm oil diet, greatly reduced the concentrations of VLDL + LDL-phospholipids. Among the mice fed polyunsaturated fat diets, a significant difference was only observed between mice fed a diet containing 100 g/kg of safflower oil and those fed a diet containing 35 g/kg of GLA oil and 65 g/kg of safflower oil in combination. The value was significantly higher in the former than in the latter. Serum free fatty acid levels were lower in mice fed diets containing 70 g/kg and 100 g/kg of GLA oil than in those fed a palm oil diet.

In relation to the observed serum lipid concentrations, we measured hepatic mRNA levels of apolipoprotein AI, a major apolipoprotein of HDL. The values were 1.00 ± 0.04 and 0.862 ± 0.067 for mice fed diets containing palm or safflower oil as a sole fat source, respectively. The levels were slightly but significantly lower in mice fed diets containing varying concentrations of GLA oil (0.808 ± 0.026, 0.770 ± 0.030, and 0.807 ± 0.040 for mice fed diets containing 35, 70, and 100 g/kg of GLA oil, respectively) than in those fed a palm oil diet. However, these values were not significantly different from the value in mice fed a diet containing safflower oil as a sole fat source.

The diets containing 35–100 g/kg of GLA oil, compared with a palm oil diet, significantly decreased the hepatic triacylglycerol concentrations, whereas a diet containing 100 g/kg of safflower oil as the sole fat source did not. However, no significant differences were observed in this parameter among the four groups of mice fed polyunsaturated fat diets. A diet containing 100 g/kg of safflower oil, compared with a palm oil diet, did not affect hepatic concentrations of cholesterol or phospholipid. All the diets containing varying concentrations of GLA oil, compared with diets containing palm or safflower oil as a sole fat source, significantly reduced hepatic concentrations of cholesterol and increased the values of phospholipids. However, these values were similar among mice fed the three diets containing different levels of GLA oil.

## Discussion

### Effects of GLA oil on hepatic fatty acid oxidation and carnitine metabolism

Previous studies demonstrated that fungal oil^(1)^ and borage oil^(2,3)^ rich in GLA increased the activity and mRNA levels of enzymes involved in hepatic fatty acid oxidation in rats (Wistar or Sprague-Dawley strain). GLA oil greatly increased hepatic fatty acid oxidation in *Apoe*^*shl*^ mice in the present study. The changes were considerably exaggerated with *Apoe*^*shl*^ mice in the present study than with rats in the previous studies. For example, a diet containing 100 g/kg of GLA oil, compared with diets containing palm or safflower oil as a sole fat source, increased the peroxisomal fatty acid oxidation rate by 5.1–5.3-fold in the present study. The effects were markedly attenuated in the previous study using Wistar rats,^(1)^ in which a fungal oil containing 25.3% GLA at a dietary level of 40 g/kg compared with soybean oil increased the value by 1.3-fold. In an experiment using Sprague-Dawley rats, borage oil containing 25.7% GLA at a dietary level of 150 g/kg, compared with palm and safflower oils, increased the value by 1.3- to 1.4-fold.^(2)^ In another experiment using Sprague-Dawley rats and borage oil containing 24.6% GLA as well as GLA-enriched borage oil containing 46.5% GLA at a dietary level of 200 g/kg, compared with safflower oil, increased peroxisomal fatty acid oxidation rats by 1.2–1.4-fold.^(3)^ Therefore, the sensitivity of the hepatic fatty acid oxidation pathway to dietary GLA may be considerably different between mice and rats. Alternatively, the fatty acid oxidation pathway in *Apoe*^*shl*^ mice compared with in other mouse strains may have a unique propensity to respond to dietary GLA. As experimental conditions differed considerably among these studies, careful examinations are still required to draw a definite conclusion. Dietary GLA not only increased the peroxisomal fatty acid oxidation rate but also enhanced the activities of various enzymes involved in fatty acid oxidation in the present study. A previous study using rats as experimental animals reported that dietary GLA increased mRNA levels of peroxisomal enzymes but did not affect those of mitochondrial enzymes.^(3)^ However, in the present study, dietary GLA greatly increased the mRNA levels of peroxisomal and mitochondrial enzymes involved in fatty acid oxidation in *Apoe*^*shl*^ mice. Peroxisome proliferator-activated receptor α (PPARα) is known to be involved in regulating gene expression of hepatic fatty acid oxidation enzymes.^(26)^ Dietary GLA oil conceivably up-regulated hepatic fatty acid oxidation through the activation of PPARα. Indeed, GLA oil not only increased the mRNA expression of mitochondrial and peroxisomal enzymes involved in hepatic β-oxidation, but also increased mRNA levels of microsomal cyp4a10 involved in ω-oxidation of fatty acids and of plasma membrane CD36 molecule, which plays a role in the cellular uptake of fatty acids from the blood circulation. Available evidence^(27,28)^ indicated that these genes are also targeted by PPARα to increase their expression.

We found that GLA oil increased hepatic concentrations of carnitine. As changes in carnitine concentrations paralleled those in carnitine transporter mRNA levels, increased uptake from the blood stream may be the primary cause for changes in hepatic carnitine concentrations. In addition, GLA oil, compared with palm and safflower oils, moderately but significantly increased the mRNA levels of enzymes involved in carnitine biosynthesis. Therefore, changes in carnitine biosynthesis may also be involved in GLA oil-dependent changes in hepatic carnitine concentrations. Previous studies suggested that PPARα also controls gene expression of the carnitine transporter^(29)^ and enzymes involved in carnitine biosynthesis.^(30,31)^

### Effects of GLA oil on hepatic fatty acid synthesis

In the present study, safflower oil and GLA oil, as well as mixtures of these oils, compared with palm oil, decreased the activities and mRNA levels of fatty acid synthase, ATP-citrate lyase and glucose 6-phosphate dehydrogenase. Both safflower and GLA oils, compared with palm oil, lowered pyruvate kinase activity and mRNA levels of Pklr. GLA oil was more effective than safflower oil in reducing these values. Moreover, GLA oil relative to safflower oil increased the activity and mRNA levels of malic enzyme, and increased mRNA levels of stearoyl-CoA desaturase 1. Similar changes were also observed in mRNA levels of fatty acid desaturase 1 and 2 involved in the metabolism of PUFAs. These conflicting results may be due to the combined effects of signals mediated by several transcription factors. The transcription factors SREBP-1c and ChREBP are master regulators of hepatic lipogenesis.^(32)^ In addition, PPARα targets some genes involved in lipogenesis and fatty acid desaturation. Regarding the role of SREBP-1 in the PUFA-mediated suppression of hepatic lipogenesis, previous studies reported that dietary PUFAs decreased the nuclear content of the mature form of this transcription factor.^(33)^ However, this was not necessarily accompanied by a decrease in the mRNA expression of SREBP-1. Although dietary PUFA-dependent changes were not observed in the mRNA levels of SREBP-1 in the present study, diets containing safflower and/or GLA oil may have decreased the nuclear content of the mature form of this transcription factor, and hence decreased the mRNA and activity levels of lipogenic enzymes. There are two isoforms of ChREBP, α and β,^(32)^ and ChREBPα is mainly localized in the cytosol. Upon stimulation, cytosolic ChREBPα is translocated into the nucleus, and up-regulates genes involved in glucose metabolism and lipogenesis. ChREBPα also targets the ChREBPβ gene to stimulate its expression. Transcriptional activity to stimulate gene expression of enzymes involved in glucose metabolism and lipogenesis is much higher with the β isoform than with the α isoform. In the present study, diets containing PUFAs, compared with palm oil, decreased the mRNA expression of ChREBPβ. This also likely contributed to the PUFA-dependent decrease in lipogenesis. ChREBP but not SREBP-1 targets the Pklr gene encoding liver-type pyruvate kinase.^(32)^ Therefore, down-regulation of the ChREBP signaling pathway may account for reductions in the activity and mRNA levels of pyruvate kinase by diets rich in PUFAs.

In addition to these transcription factors, PPARα not only affects the expression of genes involved in fatty acid oxidation, but also those related to lipogenesis. Previous studies indicated that the activation of PPARα is associated with the down-regulated expression of the Pklr gene.^(34)^ Dietary GLA oil may strongly activate PPARα, as reflected by the enhanced mRNA expression of its targets. GLA oil, not only compared with palm oil but also with safflower oil, decreased the pyruvate kinase activity and gene expression of pyruvate kinase. Both down-regulation of the ChREBP signaling pathway and up-regulation of the PPARα signaling pathway may account for GLA oil-dependent marked decreases in pyruvate kinase activities and mRNA levels. Not only SREBP-1c and ChREBP but also PPARα target the genes for malic enzyme 1^(35)^ and stearoyl-CoA desaturase 1^(36)^ to increase their expression. In addition, fatty acid desaturase 1 and 2 genes^(37)^ are also dually regulated by both SREBP-1c and PPARα, although information regarding the role of ChREBP in affecting expression of these genes is not available. GLA oil may have down-regulated SREBP-1c and ChREBP signaling pathways, but up-regulated the PPARα signaling pathway, and hence induced peculiar changes in mRNA expression of these genes.

### Effects of GLA oil on serum and hepatic lipid concentrations

Alterations in hepatic fatty acid synthesis^(38)^ and oxidation^(39)^ modify the availability of fatty acids for the synthesis of triacylglycerol, and in turn, alter VLDL production by the liver. Therefore, a change in the rate of these metabolic processes is crucial in determining serum lipid concentrations. Indeed, alterations in hepatic fatty acid synthesis and oxidation by dietary fats were accompanied by marked changes in serum lipid profiles in the current study. Noticeably, the large increases in hepatic fatty acid oxidation together with the decreases in the parameters for lipogenesis caused by the diets containing GLA oil accompanied marked decreases in serum triacylglycerol and cholesterol levels. We also confirmed that changes in cholesterol levels were ascribable to changes in the serum concentrations of apolipoprotein B-containing lipoproteins. Therefore, it is plausible that the GLA oil markedly reduced the assembly and production of apolipoprotein B-containing VLDL. As the activity and mRNA levels of lipogenic enzymes, except for malic enzyme and pyruvate kinase, were comparable between mice fed safflower or GLA oil, greater reductions in serum triacylglycerol and cholesterol levels in animals fed GLA oil than in those fed safflower oil are mainly ascribable to the promotion of hepatic fatty acid oxidation in mice fed GLA oil. As expected, alterations in fatty acid metabolism by dietary fats also accompanied changes in hepatic lipid levels. Accordingly, oils rich in PUFAs, compared with palm oil, lowered the hepatic triacylglycerol level. Although the differences were not significant, the values were lower in mice fed diets containing GLA oil than in those fed a 100 g/kg safflower oil diet. As the diets containing GLA oil, compared with diets containing either 100 g/kg of palm or safflower oil, decreased the hepatic concentration of cholesterol, GLA oil may alter the hepatic cholesterol metabolism. The diets containing GLA oil, compared with palm and safflower oils, increased liver weight accompanying the elevation of hepatic phospholipid levels, which may reflect the proliferation of mitochondria and peroxisomes. Previous studies demonstrated that agonists of PPARα increased hepatic phospholipid levels.^(40)^

We also found that GLA oil, compared with both palm and safflower oils, increased serum cholesterol and phospholipid levels in the HDL fraction, indicating that GLA oil facilitates the reverse cholesterol transport system. Therefore, GLA oil consumption induces physiological changes meritorious in preventing arteriosclerosis. However, this was rather unexpected because the present study indicated that dietary GLA oil strongly activated PPARα. Agonists of PPARα were reported to increase HDL formation through the enhanced expression of apolipoproteins AI and Aα in humans. However, a PPAR agonist (fenofibrate) as well as fish oil (an oil to activate PPARα) caused an approximately 30% decrease in hepatic apolipoprotein AI mRNA levels in mice.^(41)^ Consistent with this, we observed that GLA oil, compared with palm oil, slightly but significantly decreased hepatic apolipoprotein AI mRNA levels in the present study. Further studies to examine the effects of GLA oil on HDL formation and metabolism are required to clarify the mechanisms underlying GLA oil-dependent changes in HDL-lipid levels.

In conclusion, dietary oil rich in GLA, compared with saturated fat (palm oil) and an oil rich in linoleic acid (safflower oil), strongly increased hepatic fatty acid oxidation in hyperlipidemic mice deficient in apoE expression. Oils containing high levels of PUFAs compared with palm oil reduced the activity and mRNA levels of hepatic lipogenic enzymes. However, in general, GLA and safflower oils were comparable in affecting these parameters. GLA oil, compared with both palm and safflower oils, decreased serum lipid concentrations which may be due to alterations of hepatic fatty acid oxidation and synthesis.

## Author Contributions

TI: study concept and design; acquisition of data; analysis and interpretation of data; drafting of the manuscript; obtained funding.

IO: acquisition of data; analysis and interpretation of data.

## Figures and Tables

**Table 1:** Fatty acid compositions of dietary fats

Fatty acids (g/100 g total fatty acid)	Dietary fats		
	Palm oil	Safflower oil	GLA oil
10:0	0.2	0.0	0.0
12:0	0.7	0.0	0.0
16:0	44.0	5.2	4.1
18:0	1.2	1.2	1.3
18:1 *n*-9	39.3	9.5	5.6
18:2 *n*-6	14.7	84.1	46.4
18:3 *n*-6	0.0	0.0	42.6

**Table 2:** Effects of dietary fat on growth parameters and liver weight

	Dietary fat (100 g/kg)				
	Palm oil	Safflower oil:GLA oil			
		100:0	65:35	30:70	0:100
Food intake (g/day)	4.68 ± 0.10	4.80 ± 0.16	4.50 ± 0.07	4.48 ± 0.15	4.40 ± 0.13
Body weight (g)					
0 day	22.6 ± 0.5	22.7 ± 0.5	22.7 ± 0.6	23.0 ± 0.7	22.8 ± 0.8
18 day	27.4 ± 0.5	27.7 ± 0.9	28.3 ± 0.7	28.2 ± 0.8	27.8 ± 0.5
Growth (g/20 days)	4.8 ± 0.2	5.0 ± 0.6	5.5 ± 0.5	5.2 ± 0.5	5.0 ± 0.5
Liver weight (g/100 g body weight)	6.13 ± 0.13	6.13 ± 0.18	6.42*^,#^ ± 0.1	7.09*^,#^ ± 0.1	7.62*^,#^ ± 0.2

Values are mean ± SEM, *n* = 7. *p<0.05 vs mice fed 100 g/kg palm oil diet. ^#^*p*<0.05 vs mice fed 100 g/kg safflower oil diet.

**Table 3:** Effects of dietary fat on the activities of lipogenic enzymes

	Dietary fat (100 g/kg)				
Enzymes	Palm oil	Safflower oil:GLA oil			
		100:0	65:35	30:70	0:100
Fatty acid synthase	36.7 ± 2.5	20.4* ± 1.4	18.9* ± 2.3	20.1* ± 1.3	19.9* ± 1.4
ATP-Citrate lyase	32.1 ± 3.2	13.7* ± 1.5	10.8* ± 1.2	10.3* ± 0.6	9.27* ± 0.75
Glucose 6-phosphate dehydrogenase	11.9 ± 0.8	7.14* ± 0.61	6.89* ± 0.30	6.54* ± 0.12	6.23* ± 0.34
Malic enzyme	301 ± 21	180* ± 16	195* ± 13	245 ± 23	313^#^ ± 19
Pyruvate kinase	742 ± 46	560* ± 39	365*^,#^ ± 11	256*^,#^ ± 15	247*^,#^ ± 7

Values are mean ± SEM, *n* = 7. *p<0.05 vs mice fed 100 g/kg palm oil diet. ^#^*p*<0.05 vs mice fed 100 g/kg safflower oil diet.

**Table 4:** Effects of dietary fat on the mRNA levels of genes related to lipogenesis

	Dietary fat (100 g/kg)				
Genes	Palm oil	Safflower oil:GLA oil			
		100:0	65:35	30:70	0:100
Acetyl-CoA carboxylase α	1.00 ± 0.07	0.659* ± 0.063	0.688* ± 0.029	0.753* ± 0.047	0.624* ± 0.032
Fatty acid synthase	1.00 ± 0.08	0.476* ± 0.067	0.545* ± 0.053	0.636* ± 0.042	0.534* ± 0.057
ATP citrate lyase	1.00 ± 0.09	0.693* ± 0.094	0.568* ± 0.049	0.673* ± 0.063	0.539* ± 0.048
Glucose-6-phosphate dehydrogenase X-linked	1.00 ± 0.09	0.638* ± 0.061	0.599* ± 0.035	0.574* ± 0.015	0.602* ± 0.039
Malic enzyme 1	1.00 ± 0.09	0.621* ± 0.067	0.731 ± 0.050	1.03^#^ ± 0.11	1.10^#^ ± 0.08
Pyruvate kinase liver and red blood cell	1.00 ± 0.06	0.701* ± 0.081	0.467*^,#^ ± 0.042	0.394*^,#^ ± 0.024	0.330*^,#^ ± 0.025
Stearoyl-CoA desaturase 1	1.00 ± 0.16	0.249* ± 0.055	0.261* ± 0.031	0.673^#^ ± 0.090	0.689^#^ ± 0.092
Fatty acid desaturase 1	1.00 ± 0.09	0.659* ± 0.044	0.822 ± 0.036	1.23^#^ ± 0.050	1.19^#^ ± 0.070
Fatty acid desaturase 2	1.00 ± 0.06	0.593* ± 0.036	0.902 ± 0.063	1.33*^,#^ ± 0.080	1.11^#^ ± 0.07
SREBP-1c	1.00 ± 0.13	0.847 ± 0.192	0.720 ± 0.060	0.911 ± 0.104	0.837 ± 0.096
ChRBPα	1.00 ± 0.04	0.949 ± 0.060	0.989 ± 0.077	1.01 ± 0.07	0.937 ± 0.060
ChRBPβ	1.00 ± 0.06	0.619* ± 0.035	0.548* ± 0.042	0.549* ± 0.066	0.465* ± 0.039

mRNA levels are expressed as a fold change, assigning a value of 1 for mice fed a diet containing 100 g/kg of palm oil. Values are mean ± SEM, *n* = 7. *p<0.05 vs mice fed 100 g/kg palm oil diet. ^#^*p*<0.05 vs mice fed 100 g/kg safflower oil diet.

**Table 5:** Effects of dietary fat on the lipid levels in the serum and the liver

	Dietary fat (100 g/kg)				
	Palm oil	Safflower oil:GLA oil			
		100:0	65:35	30:70	0:100
Serum lipids (µmol/dl)					
Triacylglycerol	374 ± 27	238* ± 18	185* ± 16	157*^,#^ ± 9	161*^,#^ ± 6
Cholesterol	1,309 ± 90	691* ± 28	546*^,#^ ± 17	510*^,#^ ± 20	511*^,#^ ± 27
HDL-cholesterol	173 ± 9	159 ± 9	211*^,#^ ± 9	256*^,#^ ± 8	230*^,#^ ± 9
VLDL + LDL-cholesterol	1,136 ± 91	532* ± 26	335*^,#^ ± 14	254*^,#^ ± 13	281*^,#^ ± 28
Phospholipid	488 ± 14	278* ± 5	273* ± 9	321* ± 13	328* ± 12
HDL-phospholipid	143 ± 8	129 ± 7	175*^,#^ ± 6	218*^,#^ ± 3	198*^,#^ ± 4
VLDL + LDL-phospholipid	345 ± 17	149* ± 4	98.5*^,#^ ± 11	104* ± 11	130* ± 14
Free fatty acids	150 ± 8	106 ± 2	118 ± 9	98.9* ± 9.3	90.4* ± 6
Liver lipids (µmol/g)					
Triacylglycerol	61.1 ± 7.6	43.4 ± 5.5	39.8* ± 4.5	35.0* ± 3.3	33.7* ± 3.2
Cholesterol	6.88 ± 0.46	6.24 ± 0.27	4.28*^,#^ ± 0.16	3.58*^,#^ ± 0.12	3.69*^,#^ ± 0.14
Phospholipid	38.3 ± 0.7	40.5 ± 0.8	45.7*^,#^ ± 1.4	45.7*^,#^ ± 1.1	46.9*^,#^ ± 0.8

Values are mean ± SEM,* n* = 7. *p<0.05 vs mice fed 100 g/kg palm oil diet. ^#^*p*<0.05 vs mice fed 100 g/kg safflower oil diet.

## Abbreviations

apoE: apolipoprotein E
ChREBP: carbohydrate response element binding protein
DHA: docosahexaenoic acid
EPA: eicosapentaenoic acid
GLA: γ-linolenic acid
G6pdx: glucose-6-phosphate dehydrogenase X-linked
HDL: high-density lipoprotein
LDL: low-density lipoprotein
PPARα: peroxisome proliferator-activated receptor α
Pklr: Pyruvate kinase liver and red blood cell
PUFA: polyunsaturated fatty acid
SREBP: sterol regulatory element binding protein
VLDL: very low-density lipoprotein
